# All together now: Random Forests analysis reveals the joint impact of multiple statistical regularities on eye-movements during reading

**DOI:** 10.3758/s13423-025-02829-9

**Published:** 2025-12-16

**Authors:** Inbal Kimchi, Noam Siegelman

**Affiliations:** 1 Jack, Joseph and Morton Mandel School for Advanced Studies in the Humanities, The Hebrew University of Jerusalem, Jerusalem, Israel; 2 Department of Psychology and Department of Cognitive and Brain Sciences, The Hebrew University of Jerusalem, Mount Scopus Campus, 9190501 Jerusalem, Israel

**Keywords:** Reading, Statistical learning, Random Forests, Eye movements

## Abstract

A large and growing number of recent studies has embraced a statistical learning view of reading, revealing that readers utilize an array of regularities that are available in writing systems as they process printed words and texts. However, previous studies have focused on the impact of one regularity (or an otherwise small number of cues). Therefore, we currently have a limited understanding of (1) whether different regularities each carry unique explanatory power, beyond other (collinear) cues; (2) how do regularities at different levels of the input contribute to reading behavior; and (3) whether regularities vary in their contributions across processing stages. To answer these questions, we employ Random Forests analyses on a large-scale, eye-movement, passage-reading database from English first- and second-language readers, evaluating the relative importance of a large number of regularities on multiple eye-movement dependent variables. First, our findings demonstrate that, each regularity uniquely contributes to the model’s performance. Second, we show that both text-level regularities (e.g., predictability) and word-level regularities (including print-speech and print-meaning regularities), contribute to continuous text reading. Third, we document varying contributions of some regularities over time, with later reading measures being more impacted by text-level regularities. These results support and extend statistical learning theories of reading, showing that readers are attuned to a range of regularities in their writing system, which jointly guide naturalistic reading behavior.

## Introduction

Recent research on reading has increasingly embraced a statistical learning perspective, according to which proficient reading requires assimilating an array of patterns within the written language across different input levels (Arciuli, 2018; Frost, 2012; Sawi & Rueckl, 2019). A primary objective of research following this theoretical view, therefore, has been to identify the available regularities that exist in writing systems and investigate how they impact reading behavior.

### Statistical regularities in writing systems

The statistical cues found in print can be categorized into two groups. The first encompasses characteristics intrinsic to individual words (henceforth: word-level regularities). Much of the research on these regularities has been influenced by the triangle model of reading, where forming connections between words’ orthographic, phonological, and semantic forms is an integral part of learning to read (Plaut et al., 1996; Seidenberg & McClelland, 1989). Grounded in this view, studies have shown that measures of the degree of regularity between the “nodes” of the triangle impact reading. These include orthographic-to-phonologic (O-P) regularities, which impact performance in visual word recognition tasks (e.g., lexical decision and naming), with words more regular in O-P recognized more quickly and accurately (e.g., Baron & Strawson, 1976; Ehri, 2005; Jared et al., 1990). Similarly, words more regular in terms of their mapping between orthography-to-semantics (O-S) are recognized faster and more accurately, with studies focusing both on morphological relations (Rastle et al., 2000; Seidenberg & Gonnerman, 2000), and the systematicity between orthographic forms and meaning more broadly (Amenta et al., 2023; Marelli et al., 2015; Siegelman, Rueckl, et al., 2022a, 2022b). Furthermore, there is evidence that the degree to which a word’s spoken form predicts its orthography (i.e., P-O regularities, reflecting speech-to-print “feedback”; e.g., Perry, 2003), as well as the systematicity between spoken forms and meaning (P-S regularities; Amenta et al., 2017), impact visual word recognition.

In addition to these word-level regularities, a second group of regularities are those available in continuous texts pertaining to the relations between a word and its surrounding printed units (henceforth: text-level regularities). A notable example is *word predictability*, commonly defined as the probability of an upcoming word given its preceding context (i.e., p(word | context); see Staub, 2015). Studies have used various methods to quantify word predictability under this definition, from classic cloze predictability tasks (e.g., Lowder et al., 2018; Rayner & Well, 1996; Taylor, 1953) to modern large language models (e.g., Cevoli et al., 2022; Hofmann et al., 2022), showing that more predictable words have shorter fixations and higher skipping rates. Further, language models have also been used to assess specifically words’ semantic predictability, quantified as the similarity (e.g., cosine) between the vectorial representations of the word and its preceding context (e.g., Bianchi et al., 2020). Both general predictability and semantic predictability have been tied to reading behavior, with the latter thought of as more specific to semantic relations while the former spanning regularities at various levels (see Salicchi et al., 2023).

Another text-level regularity is *word informativeness* or *centrality*, which is the importance of a smaller linguistic unit (e.g., word) to the meaning of the larger unit in which it appears (e.g., sentence or passage). Here, too, several methods measure a unit’s importance (from subjective ratings; e.g., Yeari et al., 2015, to distributional semantics, Fan & Reilly, 2020; Kimchi et al., 2025), with less informative words shown to have less and shorter fixations.

## Examining the impact of various statistical regularities on text reading

Although the studies presented in the previous section have been highly instrumental in mapping the regularities that impact reading, they suffer from an important limitation—namely, each of these studies has focused only on one or an otherwise small number of cues. What is missing, therefore, is a holistic investigation of how various regularities *jointly* impact reading, and what is each regularity’s *relative* contribution. Such exploration is theoretically crucial, because writing systems contain a myriad of regularities, and proficient reading is presumably tied to an efficient utilization of this rich statistical information (see Siegelman, Rueckl, et al., 2020a, 2020b; Treiman & Kessler, 2022).

Such investigation, however, requires a shift in the statistical methods employed. To date, most studies in the field have employed regression-based methods (e.g., mixed-effect models). While these models are well established, they do not deal well with cases of *collinearity among predictors*, which inflates the variance of coefficient estimates, making them unstable and difficult to interpret, and complicating the identification of each predictor’s contribution. As different regularities in texts are often colinear (e.g., more predictable words are also more frequent; Bianchi et al., 2020; Shain, 2024; words higher on O-P are also higher on P-O; Siegelman, Kearns, et al., 2020a, 2020b; Chee et al., 2020), regression-based approaches are not a good method for investigating their joint and relative impact.

Instead, in the current work we use the Random Forests technique, which is better suited for handling collinear predictors, as outlined below. Random Forests are based on decision trees, a nonparametric technique, which uses a supervised learning algorithm that partitions data based on predictor variables and their relation to the outcome variable, making observations within partitions increasingly homogeneous. However, as decision trees often suffer from overfitting, Random Forests provide more generalizable outcomes: Multiple decision trees are built using bootstrapped random samples, ensuring that each tree is trained on a different subset of the data, reducing the redundancy among correlated predictors. Additionally, each tree in the “Forest” considers only a randomly sampled subset of predictors at each split, preventing highly correlated variables from dominating the model. Then, by aggregating predictions across trees, Random Forests provide relative importance estimates for each predictor: The average change in prediction accuracy across trees, beyond the other predictors in the model, reflecting a predictor’s unique contribution (Breiman, 2001; Matsuki et al., 2016; Strobl et al., 2009; Tagliamonte & Baayen, 2012).

Another crucial feature of the Random Forests technique is that it is sensitive to complex predictive patterns, providing an estimate of the *full* explanatory power of each predictor. Hence, estimated importance values from Random Forests can be thought of as capturing a predictor’s impact both in terms of its main effects and its interactions with other predictors, and both in terms of its linear and nonlinear effects (see, e.g., Auret & Aldrich, 2012; Kuperman et al., 2018; Strobl et al., 2009). As such, importance scores provided by Random Forests provide a “noise ceiling” that reflects the total role a variable has in predicting a given outcome.

Indeed, a study by Kuperman et al. (2018) demonstrated how Random Forests can be used in reading research. In their work, Kuperman et al. (2018) fitted a Random Forests model to eye-movement passage reading data, to estimate the relative importance of both basic psycholinguistic item-level variables (e.g., word length, frequency, bigram frequency) and participant-level characteristics (e.g., vocabulary, decoding, and print exposure), in predicting early and later processing stages. Kuperman et al.’s (2018) analysis both confirmed previous findings (e.g., dissociations in predictors of spatial and temporal measures), and further revealed how reader-level variables mostly affect early eye-movement measures, while text-level variables dominate later stages. More recently, Kuperman et al. (2025) further used Random Forests to evaluate the relative importance of global measures of syntactic (e.g., dependency depth, embeddedness) and lexical (e.g., type-token and noun-verb ratios) complexity in predicting eye-movement behavior. They found that reading is more strongly influenced by simple structural text properties (i.e., sentence length and position) than by syntactic or lexical complexity.

## Revealing the impact of statistical regularities using Random Forests

In line with Kuperman et al. (2018, 2025), we employ a Random Forests model to eye-movement passage reading data, to assess the relative importance of various predictors. However, our focus is on the role of the statistical regularities reviewed in the first part of the Introduction – both at the word- and text-level – in explaining different aspects of text reading behavior. Specifically, by applying the Random Forests analysis to a large-scale eye-movement passage reading of L1 and L2 data, we tackle three theoretical questions.

First, we ask whether each statistical regularity possesses independent predictive value. As noted above, since different regularities are colinear in natural texts, and given extant studies’ tendency to examine them in isolation, their unique contributions remain unclear. Our work addresses this issue by conducting an analysis with an array of statistical properties, to examine whether previously mapped regularities have nonoverlapping value in predicting reading behavior.

Second, we ask whether and to what extent word-level regularities contribute to continuous text reading. Historically, reading research has been divided into two separate literatures with limited crosstalk: one focusing on single word recognition, and the other on eye-movements during naturalistic reading (see Grainger et al., 2016; Snell et al., 2018). This division has extended to statistical learning approaches to reading—studies on word-level regularities typically use word recognition paradigms, while studies on text-level regularities use eye-movement data. However, processes related to both word recognition and extraction of information from text operate in parallel during text reading (see Snell et al., 2018) and therefore understanding how word-level regularities impact reading beyond the single word is crucial. Among the word-level regularities we consider, a particularly interesting case are regularities involving phonology (O-P and P-O). The contrast between word recognition studies (that consider phonology to be a central part of word recognition, e.g., Frost, 1998; Rueckl et al., 2015) and eye-movement studies (which rarely consider O-P relations) is perhaps most striking. We ask: Do O-P regularities contribute uniquely to text reading, and if so, what is their importance relative to other regularities?

Third, we map the contribution of different regularities to different stages of the reading process. We do so by examining how the relative importance of different regularities compares across dependent measures reflecting different processing stages: That is, whether each regularity impacts different eye-movement variables similarly, or if its influence varies across measures. Indeed, some cases of differential contribution to early versus late processing stages have been reported (see, e.g., Kimchi et al., 2025; Staub, 2015, for discussions in the context of informativeness and predictability effects). Our investigation of multiple jointly considered regularities extends this investigation substantially.

Lastly, as a secondary goal of this work, we examine these three central questions in the context of both first-language (L1) and second-language (L2) readers of the same target language, English, given suggested differences in reliance on statistical regularities between these two populations (e.g., Brice et al., 2022; Chang et al., 2019). Practically, we do so by utilizing publicly available English text reading data from a recent international collaborative project, the Multilingual Eye-tracking Corpus (MECO; Kuperman et al., 2023; Siegelman, Schroeder, et al., 2022a, 2022b). We start our investigation by looking at the behavior of L2 readers of English, due to the large L2 dataset available in MECO, which helps us validate the Random Forests approach and provide answers to our theoretical questions in a highly-powered sample. Then, we apply the same analyses on the smaller sample of L1 participants reading the same texts, and revisit the answers to the same theoretical questions in the context of L1 reading.

## Methods

### Participants, materials, and procedure

We utilized the first wave data of the MECO-L2 release (Kuperman et al., 2023; release 2.0), which includes a total of *N* = 543 participants collected in 12 testing sites. The main task in the MECO-L2 battery is an eye-tracking passage reading task conducted in English, serving as the second language (L2) for participants in 11 out of the 12 testing sites, and as a first language (L1) in one testing site (in Canada). The reading task included 12 expository texts similar to those used in English placements exams in Canadian universities, each followed by two multiple-choice comprehension questions. The texts cover encyclopedic topics like historical figures (e.g., Samuel Morse) and natural phenomena (e.g., Da Vinci’s inventions). Data from this eye-tracking task was used in our Random Forests analyses, below.

As noted above, the first (central) analysis below considers L2 readers only, due to the larger available sample size, with a sample of *N* = 498 participants. Then, we report a similar analysis on the smaller sample of L1 readers (*N* = 45). Note that L1 and L2 participants in MECO-L2 performed generally similarly in terms of comprehension accuracy (*M* = 74% vs. 72%; Cohen’s *d* = 0.13). This is despite some differences in component skill tests of English also included in MECO (spelling: *M* = 40.1 vs. 34.5; *d* = 1.3; speeded word naming: *M* = 91.5 vs. 80.0; *d* = 0.99; speeded nonword naming: *M* = 59.2 vs. 49.9; *d* = 0.69; lexical decision accuracy: *M* = 91.2% vs. 75.0%; *d* = 1.44; and vocabulary knowledge: *M* = 38.6.4 vs. 29.4; *d* = 0.96; see Kuperman et al., 2023, for details).

### Analytical approach*:* The Random Forests technique

As noted above, the Random Forests technique constructs multiple decision trees from random samples of data and predictors, then averages their predictions to improve accuracy and reduce overfitting. The Random Forests algorithm assesses the relative importance of predictors through variable permutation and model refitting. If a relative importance of a predictor is close to zero, it suggests that this predictor has little to no impact on the predictive performance of the model (i.e., the predictor does not contribute to reducing the uncertainty or improving the accuracy of the predictions made by the Random Forests). However, as the relative importance of a predictor increases, it indicates that the predictor is playing a more significant role in enhancing the model’s accuracy, providing valuable information that helps in making more precise predictions.

We used the Random Forests algorithm implemented in the “cforest” function of the *party* package in R (Hothorn et al., 2006; Strobl et al., 2007, 2008). Key parameters in applying Random Forests are the number of trees to be built (*ntree*) and the number of randomly sampled predictor variables for each split point (*mtry*). Following Kuperman et al. (2018), we set the number of trees built in each Random Forests analysis to 1,000. A common practice in Random Forests models is to set the number of randomly sampled predictors used to select each split point (the *mtry* parameter) to either the square root of the number of predictors (Breiman, 2001) or one-third of the predictors (Liaw & Wiener, 2002). To improve stability and estimate the robustness of our estimates, we follow Kuperman et al. (2018) and Genuer et al. (2010), and vary the *mtry* value between the square-root of the number of predictors (3) and one third of the number of predictors (5) in increments of 1. Thus, for each of the dependent variables that we extracted from the eye-movement record (see below), we ran three Random Forests models, with the *mtry* set to 3, 4 and 5 for each run, resulting in a total of 24 models. The results for each dependent variable were then averaged to obtain an aggregated importance estimate for each predictor in each dependent variable; we also report below the results for each *mtry* value for transparency.

### Dependent variables

In eye-movement reading research, multiple dependent variables are commonly extracted from the data, each reflecting a different aspect of reading at different processing stages (see, e.g., Godfroid, 2020; Rayner, 1998, for a detailed discussion). Here we used six central dependent variables: (1) *first run skipping*, a binary measure of whether a word was skipped or not during the initial pass; (2) *first fixation duration*, the duration of the initial fixation on the word; (3) *gaze duration*, the summed duration of fixations on the word during the initial pass; (4) *total fixation duration*, the cumulative duration of all fixations on a specific word; (5) *rereading duration*, the summed duration of all fixations on a word excluding the ones in the initial pass (i.e., the total fixation duration minus the gaze duration); and (6) *rereading*, a binary measure that indicates whether the participant’s gaze returned to a previously read word after the first pass.

### Predictors

We used 15 measures as predictors reflecting different text-specific characteristics. These included a set of eight basic psycholinguistic variables that are known to impact reading behavior, and a set of seven measures of statistical structure, including both word- and text-level regularities, which form the center of our investigation. The rationale behind including not only our central measures of statistical structure but also the known basic psycholinguistic variables was to ensure that our findings regarding the former set of measures hold when the impact of basic word properties is considered. As for the set of measures of statistical structure, we preemptively state that these are not meant to be an *exhaustive* list of all potential regularities that exist in writing systems. Rather, they are meant to represent central (word level and text level) sources of statistical information that have been suggested to impact reading processes, as reviewed in the Introduction (see more in the General Discussion).

#### Basic psycholinguistic variables

(1) *Word frequency*, frequency counts from the English Lexicon Project (Balota et al., 2007), log-transformed; (2) *mean bigram frequency*, frequency counts from the English Lexicon Project (Balota et al., 2007), log-transformed; (3) *word length* (i.e., the number of letters in the word); (4) *number of morphemes*, using the morpheme counts from the English Lexicon Project (Balota et al., 2007); (5) *number of orthographic neighbors*, defined as all words with a Levenshtein distance of one letter from the target word, taken from Siegelman, Rueckl, et al. (2022a, 2022b). For our analysis, we added one and log-transformed these values; (6) *number of phonological neighbors,* defined as all words with a Levenshtein distance of one phoneme from the target word, taken from Siegelman, Rueckl, et al. (2022a, 2022b). Again, we added one and log-transformed these values; (7) *concreteness*, the degree to which the concept denoted by a word refers to a perceptible entity, measured by mean concreteness ratings collected by Brysbaert et al. (2014); (8) *age of acquisition*, the age at which a word was learned, based on age of acquisition ratings by Kuperman et al. (2012).

#### Measures of statistical structure

1. *Orthography-to-phonology consistency* (O-P consistency) defined as the degree of consistency in the mapping from a word’s spelling to its pronunciation. We used an operational measure of feedforward body-rime O-P consistency (rime is a higher-order grouping of the vowel and the coda of a syllable, i.e., any consonants that follow the vowel; for instance, the rime of the monosyllabic word “flash”/flæʃ/is/æʃ/). A word is feedforward consistent if its pronunciation matches that of similarly spelled words. We used the values from Chee et al. (2020), using the raw feedforward rime consistency for monosyllabic words, and a composite score, i.e., the mean feedforward body-rime consistency of all syllables for multisyllabic words.
2. *Phonology-to-orthography consistency* (P-O consistency)—that is, the mapping from sound to spelling, calculated by feedback body-rime consistency. A word is feedback consistent if its spelling matches that of similar pronounced words. Again, we used the scores calculated by Chee et al. (2020), using the raw feedback body-rime consistency for monosyllabic words, and the composite score for multisyllabic words.
3. *Orthography-to-semantics consistency* (OSC), the degree of regularity in the mapping from spelling to meaning. We used a measure where OSC is calculated as the mean cosine semantic similarity between a word and its orthographic neighbors (from Siegelman, Rueckl, et al., 2022a, 2022b).
4. *Phonology-to-semantics consistency* (PSC), the regularity in the mapping from sounds to meaning (i.e., in the opposite direction of OSC), defined here as the mean cosine semantic similarity between a word and its phonological neighbors (from Siegelman, Rueckl, et al., 2022a, 2022b).
5. *XGLM predictability*, that is, the degree to which a word form is (un)predictable in a given written context. We used here the surprisal values from A. De Varda and Marelli (2023), defined as the negative logarithm of its probability conditioned by the preceding sentence context (using the XGLM Transformer-based language model; Brown et al., 2020). We have opted to use computational estimates (rather than human-based estimates from a cloze task) given the high degree of overlap between approaches (A. G. de Varda et al., 2024; Goldstein et al., 2022), and recent findings suggesting that if anything, computational approaches provide better explanatory power than human ratings (most likely due to their better coverage in lower ends of the predictability continuum; e.g., Lopes Rego et al., 2024).
6. *Semantic predictability*, following Bianchi et al. (2020), we used a definition where semantic predictability is calculated by the cosine similarity between the vector representation of the specific word and the vector representation of the 50 preceding words (using FastText word embeddings; Bojanowski et al., 2017).
7. *Word informativeness* defined as the significance or importance of a word to the meaning of the sentence in which it appears, calculated as the natural-log transformation of 1 minus the cosine similarity between the vectorial representation of the entire sentence and the vectorial representation of a revised sentence with the exclusion of that word, taken from Kimchi et al. (2025). Words that are more informative to the meaning of the full sentence thus have a higher informativeness value per this measure, which were shown to lead to longer total reading times and higher rereading rates.

### Data cleaning

We started by separating the MECO-L2 eye-movement data into two datasets: L1 readers (71,155 data points) and L2 readers (710,435 data points). We then further excluded outliers at the interest-area level by removing observations with total fixation duration shorter than 80 ms or longer than 2,000 ms, as well as interest areas with first fixation duration longer than 800 ms or gaze duration longer than 1,000 ms (all these values presumably reflect unrealistic fixation times, related to tracker failure or other data issues). This resulted in filtering ~3% of interest areas. Lastly, we excluded words with missing values in one or more of the predictors’ measures. For L2 readers, the analysis for first run skipping was based on the remaining 476,931 data points, and analyses of the remaining five eye movement measures (where skipped words were excluded) had 334,822 data points. For L1 readers, the first run skipping analysis was based on 48,257 data points, and analyses of the remaining five dependent variables were based on 28,591 data points (i.e., excluding skips).

### Evaluating model fit via cross-validation

To test the Random Forests models’ general predictive power, we only used 90% of the datapoints to fit the models, from which all importance scores below were estimated. The remainder of the data (10%) was used to evaluate the models’ fit, by computing the agreement between the predicted values based on the fitted model for the held-out data and their actual values, for each dependent variable. We further applied the same cross-validation approach using a standard linear model (or generalized linear models, in case of binary variables), to serve as a benchmark for comparison. As shown in Table 1, models’ fit was higher for the Random Forests models than for the linear models, in all dependent variables. This is expected due to Random Forests’ ability to capture various linear and nonlinear, additive and interactive, relations.

**Table 1:** Cross-validation of Random Forests models on MECO-L2 Data (L2 Readers), and comparison to standard linear models. Values show the correlation between model predictions and actual values for continuous measures, and classification accuracy for binary measures, in the 10% held-out set

Dependent variable	Random Forests	Linear model
First run skipping	67.6%	65.6%
First fixation duration	0.18	0.10
Gaze duration	0.32	0.26
Total fixation duration	0.33	0.29
Rereading duration	0.22	0.16
Rereading (binary)	75.7%	52.2%

Correlations/classification accuracy for Random Forests are the average across *mtry* values; the range across these values was always smaller than 0.01 (for correlations) or 0.06% (for classification accuracy). Linear models were based on a linear link function for numeric outcomes, with log-transformation for gaze duration and total fixation duration, and logistic models for binary outcomes.

## Results

### Collinearity among predictors

**Fig. 1:**
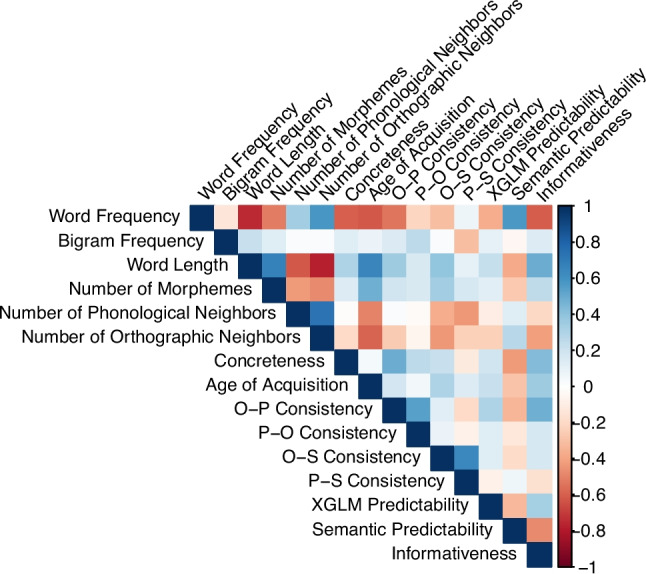
Correlations between predictors. (Color figure online)

Figure 1 presents the correlations between all predictors, revealing, as expected, substantial multicollinearity. In particular, there were notable correlations among multiple measures of statistical structure (e.g., O-S consistency and P-S consistency: *r* =.64; O-P consistency and P-O consistency: *r* =.54; semantic predictability and informativeness *r* = −.47), as well as between these measures and the basic psycholinguistic variables (e.g., frequency and semantic predictability: *r* =.58; informativeness and length: *r* =.49). This again reinforces the significance of using an approach that can better handle collinearity.

### Random Forests analysis of eye-movements in L2 readers

**Fig. 2:**
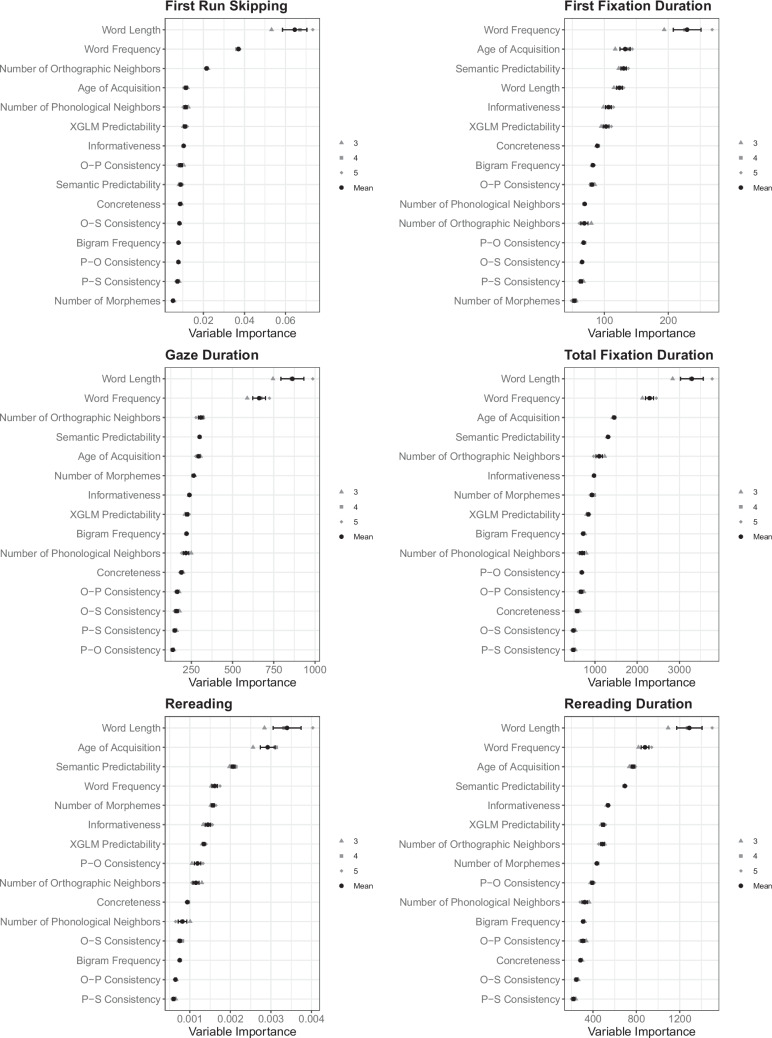
Relative variable importance of all predictors for all dependent variables in L2 readers

For each predictor and each dependent variable, the Random Forests analysis provides an estimated relative importance value for each *mtry* value, as well as a mean estimated relative importance and a corresponding standard error (across *mtry* parameters), presented in Fig. 2. These results already conceptually replicate known findings (i.e., strong effects of length and frequency across multiple dependent variables). They also point to the unique impact of various measures of statistical structure, which we discuss below.

**Fig. 3:**
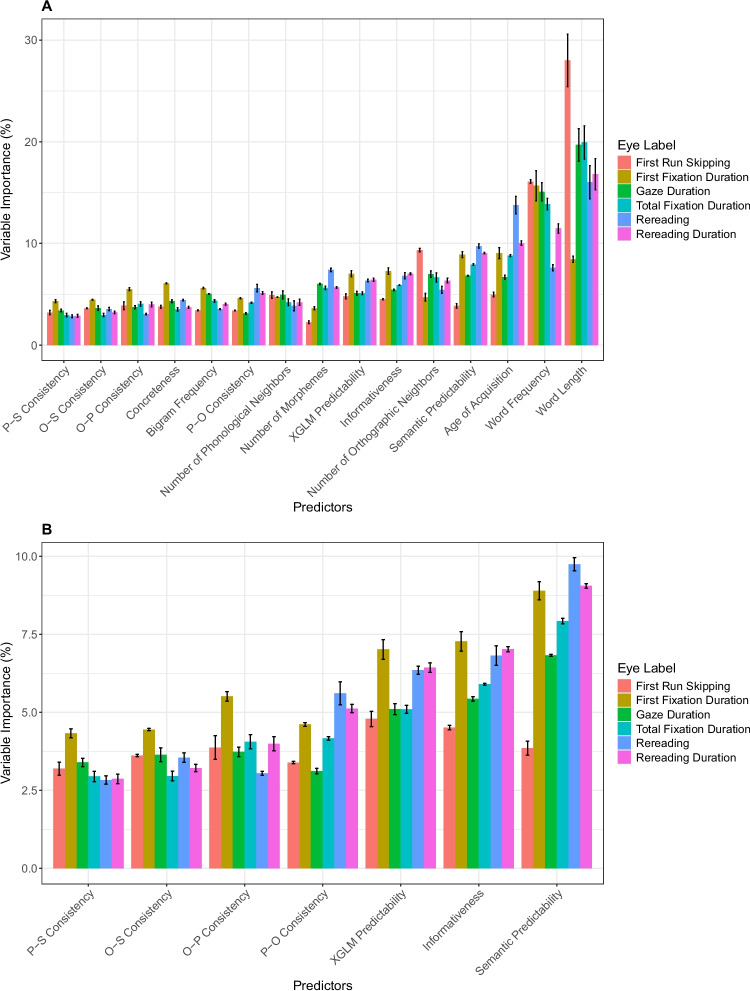
Percentage of variable importance for each predictor in each eye-movement measure in L2 readers. **A** All predictors in the model (including basic psycholinguistic variables). **B** Zoom-in on measures of statistical structure (for readability). (Color figure online)

However, Fig. 2 does not allow for *comparing* importance across different dependent variables. This is because the importance values of each predictor are influenced by the scale and the variance of the dependent variable, as well as its type (i.e., numeric vs. binary). In other words, because importance values are relative *within* each model, high values in one dependent variable do not necessarily reflect stronger effects than lower values in another dependent variable (Matsuki et al., 2016; Strobl et al., 2009). Therefore, we next computed the *percentage* of variable importance for each predictor in each dependent variable: The percentage of importance that each predictor contributes to the total importance of all predictors for a given eye-movement measure (i.e., a predictor’s relative importance divided by the sum of importance values to a dependent variable). Point estimates and standard errors for this measure for each predictor in each dependent variable are presented in Fig. 3.^1^ This normalized measure allows comparison of predictors’ importance across dependent variables, and thus to tackle the three theoretical questions from the Introduction.

#### Do different statistical regularities possess independent predictive value?

As shown in Fig. 3, the point estimates of relative importance values of all measures of statistical structure for all dependent variables are larger than zero, with little uncertainty. In other words, each predictor uniquely contributes to the model’s performance regardless of the dependent variable considered, providing evidence that all measures of statistical structure offer some explanatory power even when considered concurrently. Unsurprisingly, the proportion of relative importance associated with measures of statistical structure (ranging from 2.8% to 9.7%) is generally lower than the most well-established predictors of eye movements (e.g., values for length and frequency range between 7.6% and 28%). Still, the relative importance of measures of statistical structure is on par with other commonly studied basic psycholinguistic variables (e.g., bigram frequency, concreteness).

#### Do word-level regularities contribute to continuous text reading?

Among the measures that show above-zero relative importance are the measures of word-level regularities (i.e., O-P/P-O consistency, O-S/P-S consistency). As one might expect, word-level regularities generally have lower proportions of relative importance than text-level regularities (see Fig. 3B). Importantly, however, word-level regularities do have unique explanatory power (in a similar order of magnitude), suggesting that they play a role even when considered jointly with many other predictors in analyses of text reading. Notably, this is true—with generally comparable relative importance estimates—both for measures related to access of semantics (i.e., O-S and P-S), and measures related to phonological processing (O-P and P-O).

#### Do regularities vary in their contribution to different stages of reading?

As shown in Fig. 3B, in first fixation duration, gaze duration, and total reading time, both word-level and text-level regularities generally have a similar trajectory of relative importance in different predictors. However, late eye-movement measures (rereading and rereading duration) are more related to text-level than word-level regularities, in line with some previous findings using regression approaches (e.g., findings about informativeness in Kimchi et al., 2025). In contrast, word-level regularities seem to be less related to rereading measures, with one exception: P-O consistency showing increased relative importance in rereading measures, potentially because it reflects feedback from phonology-to-orthography which may be more slowly computed.

### Random Forests analysis of eye-movements in L1 readers

**Fig. 4:**
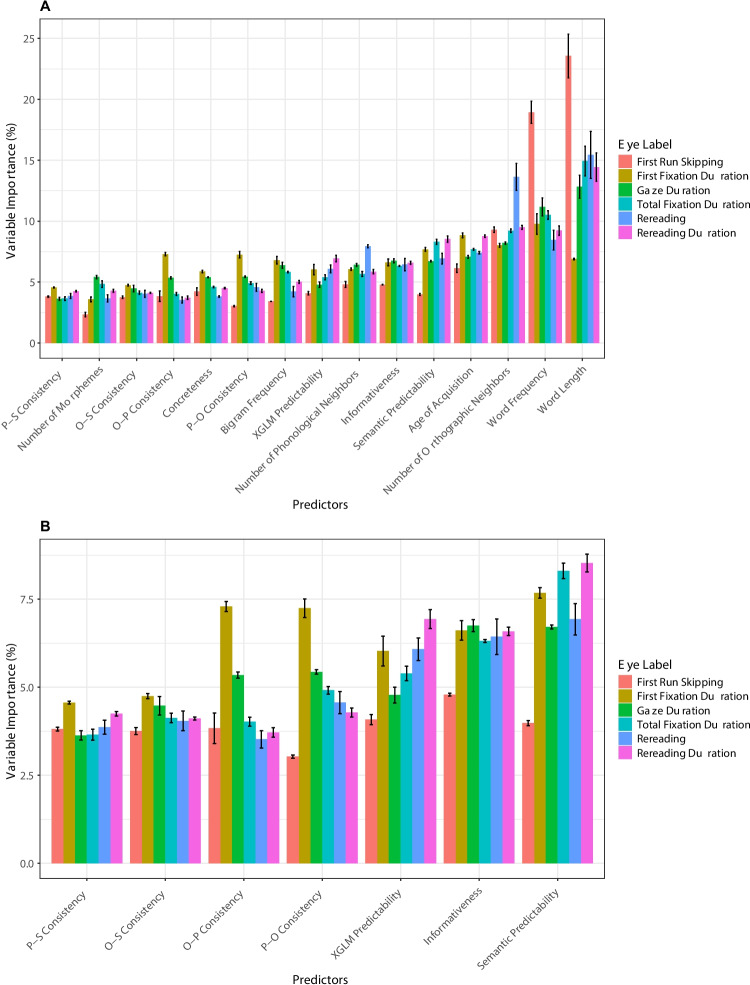
Percentage of variable importance for each predictor in each eye-movement measure in L1 readers. **A** All predictors in the model (including basic psycholinguistic variables). **B** Zoom-in on the seven measures of statistical structure (for readability). (Color figure online)

We next conducted the same analysis on the L1 dataset, to examine whether and to what extent the findings above generalize to L1 readers. The percentage of variable importance for each predictor in each dependent variable among L1 readers is shown in Fig. 4. We note that, first, as in L2 readers, all relative importance values of all measures of statistical structure in all dependent variables are larger than zero also in L1 readers, indicating that each predictor offers unique explanatory power. Second, Fig. 4B again shows unique contribution of word-level regularities to continuous text reading. Third, rereading measures seem to again be tied more to text-level than word-level regularities. However, some distinct patterns do emerge in L1 readers in terms of the reading time-course: In particular, P-O consistency, which has increased relative importance in predicting rereading in L2 readers, does not produce such trend in L1 reading. Rather, both O-P and P-O consistency have a descending pattern of importance across reading stages, with greater relative importance in *early* measures. We return to these more subtle L1–L2 differences below.

## Discussion

In this study, we employed Random Forests models on eye-movement passage reading data to evaluate the relative importance of various statistical regularities in eye-movement behavior. Our findings indicate that each predictor uniquely contributes to the model’s performance, regardless of the dependent variables considered, in both L1 and L2 readers. This suggests that various measures of statistical structure offer some degree of explanatory power even when analyzed concurrently, jointly operating in the extraction of information from print. Notably, we found that word-level regularities, including those related to phonological processing, possess unique explanatory power in text reading, although they do have generally lower relative importance than text-level regularities. We also observed varying contributions across different stages of reading, as reflected in different importance proportions for different eye-movement dependent variables (e.g., in both L1 and L2 readers, rereading was more related to text-level than word-level regularities; however, there were also L1-L2 differences in the time-course, discussed below).

Broadly, our results validate and extend statistical learning views of reading (e.g., Arciuli, 2018; Sawi & Rueckl, 2019). They show that readers are sensitive to a range of regularities in their writing system, which jointly guide reading behavior. They also show that there is no clear demarcation between word- and text-level regularities: Both sources of information explain behavior in naturalistic text reading, although the exact role each regularity plays varies as a function of the considered dependent variable. This is an important theoretical observation from a statistical learning perspective: It suggests that the significance of any given statistical regularity depends on the specific reading task and dependent variable, rather than a strict division between word- and text-level properties. This finding also has implications for computational models of reading. Historically, models of eye-movements have centered on specific psycholinguistic variables (most commonly, frequency, length, and predictability, e.g., Pollatsek et al., 2006), while the impact of word-level regularities has been the focus of word recognition models (e.g., Harm & Seidenberg, 2004; Plaut et al., 1996). Our results instead highlight the interrelated nature of these processes and the need to better integrate the two types of models (see also Siegelman et al., 2025; Snell et al., 2018).

Clearly, however, there are open questions highlighted by our results, which require further investigation. In particular, our results raise interesting questions about L1–L2 similarities and differences. On the one hand, our findings highlight broad L1–L2 similarities: In both samples, multiple regularities had a unique impact on text reading behavior, with predictive value also for word-level regularities, and rereading measures related more to text-level regularities. On the other hand, however, more subtle L1–L2 differences emerged, particularly in the impact of statistical regularities over time. Thus, whereas in L2 readers, most word-level regularities had a rather stable impact, with the exception of speech-print “feedback” consistency that had a higher impact on rereading, in L1 readers, regularities involving phonology (O-P and P-O) had greater importance in *early* stages of reading. This suggests that L1 readers may compute phonology earlier and more efficiently given their extensive experience with the spoken language (see Brice et al., 2022; Chang et al., 2019, for related discussions). While these results should be interpreted with caution given the small L1 sample, they do raise interesting future directions, suggesting that L1 and L2 readers may differ not only in the extent but also in the time-course of their utilization of statistical structure from print. Relatedly, in the current work we only examined group-level behavior, ignoring individual differences within groups. We leave it for future research to examine whether participant’s characteristics (e.g., reading skill) systematically relate to their relative “weighing” of different regularities as reflected in their relative importance.

Lastly, we highlight two other future directions. The first is the exact link between measures of statistical structure and reading behavior. Recall that unlike traditional regression-based approaches, estimated Random Forests’ importance values capture complex predictive patterns, including both linear and nonlinear effects, main effects and interactions. Thus, importance values reflect all that can be explained by a variable. Further targeted models and more theoretically-constrained approaches are required to identify the sources of these effects and assess *how* each predictor relates to reading behavior. The second question is the exact scope of regularities readers rely on during reading. Although encompassing a relatively wide range of regularities, there are other documented regularities not included in our analysis. These include, for example, the quasiregularities of word length combinations (Snell & Theeuwes, 2020), links between printed forms and phonological features such as lexical stress (Arciuli, 2018) or vowel length (Treiman et al., 2023), and additional facets of predictability (e.g., syntactic predictability, Luke & Christianson, 2016). We have also made specific assumptions in the measures that are included (e.g., using computational predictability estimates rather than human-based ratings, which may not overlap fully; see Lopes Rego et al., 2024). Still, our findings provide support for statistical learning views by showing that readers utilize a wide array of regularities to process information from printed texts. The approach laid out in the current paper can be further used to examine the exact scope (and limits) of this sensitivity.

## Data Availability

Data is publicly available via the Open Science Foundation website: https://osf.io/sbpzm.

## Footnotes

1 Percentage of variable importance should not be confused with portion of explained variance. Portion of explained variance is computed based on the total deviations and is thus impacted by unexplained error variance. Proportion of relative importance only considers predictors that *are* included in the model.

## Code availability

Code is made publicly available via the Open Science Foundation website: https://osf.io/sbpzm.
